# Alternate gene expression profiling of monoterpenes in *Hymenocrater longiflorus* as a novel pharmaceutical plant under water deficit

**DOI:** 10.1038/s41598-022-08062-x

**Published:** 2022-03-08

**Authors:** Armin Saed-Moucheshi, Ali Akbar Mozafari

**Affiliations:** Department of Horticultural Science, Faculty of Agriculture, University of Kurdistan, Sanandaj, Iran

**Keywords:** Agricultural genetics, Biotechnology, Plant biotechnology, Agricultural genetics, Plant sciences, Plant biotechnology, Plant breeding, Plant genetics, Plant physiology, Plant signalling, Plant stress responses, Secondary metabolism

## Abstract

*Hymenocrater longiflorus* (surahalala) is a wild plant species with potential pharmaceutical and ornamental interest. To date, the genomics of this plant is unknown and the gene expression profiling of the genes related to its metabolite has never been studied before. In order to study the responses of in vitro-grown surahalala plants to abiotic stresses and the differential expression of the genes related to its essential oils under exogenous proline application; three levels of PEG600 (0, 10, and 20%) and five levels of proline (0, 5, 10, 15, and 20 µm) were combined in the culture media. Thus, water deficit increased oxidants levels and decreased fresh weight of surahalala tissues, whereas addition of proline up to 15 µm was able to relatively compensate the negative effect of water deficit. Contrarily, high proline level (20 µm) had a negative effect on surahalala plants probably due to the stress simulation (nutrition) under high proline concentration. In addition, the best combination for achieving highest essential oils content was 10 µm proline plus 10% PEG. The expressional profiling of the genes *TPS27*, *L3H*, *TPS2*, *TPS1*, *OMT* and *GDH3* were successfully carried out and their involvement in 1,8-cineole, carvone, α-pinene, thymol, estragole and β-Citronellol biosynthesis, respectively, was verified. In addition, our results indicated that these genes could also be involved in the synthesis of other metabolites under water deficit condition.

## Introduction

Tissue culture is a successful method to produce plants independent of the environment which is also very applicable for growing plants with difficulties for seed production such as *Hymenocrater longiflorus* Benth. This plant is native to north-west of Iran (Kurdistan province) and north-east of Iraq (Kurdistan state) and it has been known as surahalala by natives. Surahalala could be categorized as both pharmaceutical and ornamental plants^1^. However, it is only available in its high-altitude places of origin, and has never been grown under field or greenhouse conditions by farmers due to its low seed viability and difficult germination. Therefore, studies on this plant are rare and most of them have been carried out by the institute of the authors. In a previous study^2^, a successful in vitro growth methodology was described. According to traditional pharmaceutical plant texts and pharmacists along with more recent studies^1^, surahalala possesses different pharmaceutical properties such as anti-allergic, anti-inflammatory, and sedative effects. Shahriari et al.^3^ have reported a high antioxidants activity (e.g., SOD and POD) and secondary metabolite (e.g., carvacrol and thymol) contents in its shoots, and Hoseiny et al.^2^ showed that essential oils contents in the shoots are enough to be used for pharmaceutical and essential oils extraction purposes.

According to Hoseiny et al.^2^, using any extra biochemical compounds along with the standard constituents in the culture media for growing surahalala can almost certainly stimulate or suppress the production of some essential oils. Since the quality and quantity of the essential oils produced by this plant are an important feature in its pharmaceutical potential, studying the effect of applying different compounds and substances on its essential oils content is necessary^4^. To date, to the best of our knowledge, the only study on this was carried out by Hoseiny et al.^2^, who showed that adding a mixture of salicylic acid and simvastatin (SV) in the medium markedly changed the essential oils content in surahalala. On the other hand, there are many articles indicating the crucial influence of environmental stresses on essential oils and metabolite content of different plants^5^. Langroudi et al.^6^ showed that drought stress can change essential oils contents and final production of *Rosmarinus officinalis*. Saed-Moucheshi et al.^7^ stated that drought stress is able to increase the antioxidant contents of triticale as a result of higher expression rate of related genes.

Proline is an essential compound in plants mainly under stress conditions^8^. Proline, as a free amino acid in plant cells and tissues, is mostly involved in osmotic adjustment and antioxidant activities under different stresses^9^. In addition, pharmaceutical properties and application in brewing industries have been reported for the free amino acid. Saed-Moucheshi et al.^10^ reported antioxidant activities for proline in different plant species. Different reports have verified the impact of proline as enhancer or suppressor of the expression of different genes^11^. Proline has upregulated the expression of some abscisic acid (ABA)-related genes such ABA1, ABI1 and AXR2 in Arabidopsis, which are mainly involved in stress responsive pathways^12^. There are also other reports such as Li et al.^13^, Lee et al.^14,15^, and Sofy et al.^16^ related to the interaction of proline with jasmonic and salicylic acid gene networks. Although the studies on proline application on essential oils under water deficit are rare, some studies^15,17^ showed that proline may be able to indirectly affect the molecular pathways of some metabolites.

Despite of the fact that surahalala has a great potential to be used in pharmaceutical industries, studies on the effect of different treatment on its phytochemical contents are rare. Moreover, this plant has never been studied under drought stress condition or proline treatment. Also, there is no study on the influences of drought treatment on surahalala plant. Similarly, the expression profiling of the surahalala genes involved in essential oils has never been studied. Therefore, this study aimed to consider the effects of PEG application, as drought stress simulator, and proline on the quantity and quality of some essential oils and biochemical content of surahalala. Furthermore, the expression profiling of six different genes involved in biogenesis pathways of the essential oils was carefully assessed. additionally, any possible associations of the expression rate with biochemical contents of surahalala have been considered by different methods of data mining.

## Materials and methods

### Plant materials and treatments

Plant samples of surahalala have been collected by the help of knowledgeable natives from its natural habitats in Kurdistan, Iran. Since *Hymenocrater longiflorus* Benth. is not considered as a field crop that is normally cultivated in agricultural fields, the full-grown, healthy herbaceous branches of the species were sampled from mountains in the western region of Iran, its natural habitat and origin, with geographic coordinates of 35.2526° N and 46.2612° E and altitude of 1435 m in 2021. The samples were carefully transferred to the Tissue Culture Lab of the University of Kurdistan in order to be distinguished and get used as explants in the tissue culture experiment. The final identification of the plant was done by Prof. Mozafari according to HKS-1552 and HKS1558 samples deposited in Botanical Herbarium of University of Kurdistan.

These samples were selected from the youngest, healthy, and the full-grown herbaceous shoot parts of the plants and they carefully transformed to the Biotechnology and Tissue culture lab of Horticultural Science, University of Kurdistan. After preparing the samples through washing and sanitizing they were cultured in MS^18^ medium prepared based on the instruction that was formerly introduced by the mentioned lab and is fully described in Hoseiny et al.^2^. In summary, the prepared medium was containing all necessary substances as well as 5 mg/L benzyl aminopurine (BAP) (Sigma Aldridge Company Ltd.) and 0.1 mg L^−1^ indole butyric acid (IBA) (Merck KGaA Company). After preparation of liquid medium, 7 gr agar was added so that the medium been able to change into its solid stat. before starting the main experiment, the collected samples were cultured in the mentioned medium in 250 mL volume glass container in order to produce the callus form. After that, the produced calluses were transferred to glass container containing their medium to produce the root and shoot. By the time explants produced both shoot and root, they were then transferred to other glass container with media containing the experimental treatment, polyethylene glycol (PEG) 6000 and proline (Pro). Both PEG and proline were mixed with the media culture before adding the agar and poured into the glass containers. Three levels of PEG containing 0, 10, and 20% V/W were applied to simulate the water deficit condition along with five levels of proline consisting of 0, 5, 10, 15, and 20 µm. It should be mentioned that the PEG levels were selected according to the previous experiment conducted by the authors in the same lab so that the plants continue their growth in line with being affected by the treatment’s levels. In addition, the proline levels were based on the most of applied concentration on proline in the previous studies published in the literatures related to the different plant species. Each treatment was applied in one glass container containing two explant and was repeated 8 timed (3 PEG, 5 Pro, 8 rep; 125 overall units). The glass containers were placed in a growth chamber Convivon, CMP 4030, Canada) under ultraviolet (UV) light (46 µEm^−2^ s^−1^ light intensity) light produced by fluorescent light instrument with/8 h illumination/darkness conditions along with 25 ± 1 °C temperature and 55 ± 2% relative humidity. After 35 days from starting the experiment, when plants were fully expanded, the sampling for the measurements were done.

No human subject was used in this experiment, but all the plants materials, including the collected and used plant material, completely comply with institutional, national, and international guidelines and legislation regarding this type of experiment.

### Feature measurements and essential oil components

After finalizing the experiment, the whole plants (two sub-samples or plantlets) were taken out and the root and shoot were separated from the media and were weighed using an accurate digital scale (AND Japan FX400; 0.00001 g accuracy) after removing the medium by washing with double distilled water. Shoot fresh weight (SFW) and root fresh weight (RFW) were measured and recorded for each unit and were reported as average of the two sub-samples (plantlets samples) in each glass container according to gram unit.

After weighing the shoot samples, 0.5 g of these sample were used for measuring free proline content^19^, hydrogen peroxide (H_2_O_2_) content^20^, and superoxide dismutase activity^21^ by using spectrophotometer (UV-2100 model suv, New Jersey) method.

Ten grams of the shoot samples were used for extracting the essential oils contents in three randomly selected glass container (repeat). Hydro-distillation method was applied for extracting essential oils components via using Clevenger method. The complete method of extraction is fully described in Hoseiny et al.^2^.

### Expression rate and RExpressin package development

In this study a package in R language has been developed which is able to extract the direct results of gene expression from the Ct (threshold cycle) output of both target and reference genes in real-time quantitative PCR. This package named ‘RExpressin’ its source as well as its instruction use are available online in a GitHub repository (https://github.com/ArminSaed/RExpression). Also, a copy of this package, one in ‘zip’ format for Windows users and one in ‘gz’ format for Linux users, is presented as supplementary material in this paper. The mentioned package was installed on RStudio and used for calculating the expression rate of the considered genes in this study involving in essential oils and secondary metabolite production pathways. The method of delta Ct was used for preparing the ‘RExpression’ and is described in summary as follow.

Determined Ct of target genes gene was subtracted from the Ct of reference gene as ΔCt. After that, ΔCt of each treatment level (here 3 PEG levels by 5 proline levels) was subtract from the average ΔCt of the control treatment (no PEG or proline for the current study). Finally, by assuming the complete performance of PCR, the 2^−ΔΔCt^ formula was used for relative expression rate where the rate of the control is equal to 1. In developed package, the column number of treatment variable and reference gene should be specified. The out of this package is containing the average expression rate of each treatment as well as its SE. This package is also able to draw 10 different plots for comparing the treatments and also to test the quality of the results which is specified by a number from 1 to 10.

### Quantitative RT-PCR

The Total RNA of Hymenocrater longiflorus shoots of all treated units were extracted by using RNA extraction kit (Cinapure, Cinagene, Iran) based on the manufacturer’s protocol. The quantity of RNA was considered by spectrophotometer method (Biochrom, United Kingdom). The direct electrophoresis (Bioanalyzer, Agilent, USA)) of total RNA on agarose gel was used to determine the quality of the total RNA in which the 16 s and 24 s rRNA were distinguishable. Total RNA was then transcribed into cDNA using the Reverse Transcription Kit (Cinapure, Cinagene, Iran) according to the instruction of manufacturer. In order to survey the quality of cDNA, the acting primers were used for PCR and the product was transformed to agarose gel where two bands were sharply observable.

The RT-qPCR was performed using StepOnePlus Real-Time PCR System containing 96 well (Applied Biosystems StepOnePlus™ system, USA). Three biological replicates per treatment were considered for RT-PCR. Two internal control genes (Actin and elongation factor; EF1) were applied as internal controls. The relative expressional levels of TPS27 gene, may also call 1,8-cineole synthase1 (involved in 1,8-cineole synthesis), Limonene-3-hydroxylase (L3H) gene (involved in carvone synthesis), pinene synthase (TPS2) gene (involved in α-pinene synthesis through MVA pathway), TPS1 gene (involved in thymol synthesis), OMT or O-methyltransferases gene or (involved in estragole synthesis) and GDH1 (Geraniol dehydrogenase) gene (involved in β-Citronellol synthesis). Due to the lack of genomic data of surahalala, we considered all sequence of the mentioned genes in its closet species, such as *Salvia officinalis L*, *Hybrid lavandin (L. angustifolia* × *L. latifolia*)*, Origanum vulgare*, *Melissa officinalis*, *Dracocephalum moldavica*, *Majorana hortensis syn. Origanum majorana*, etc., and after finding the similar motif by running the blast test in NCBI site, the primers were mostly designed through these similar sequences by using AlleleID software. Before using these primers in final RT-qPCR their efficiency and quality were checked by changing the temperatures of PCR stages and adding or removing one or some of the nucleus based mostly from the 5′ end (Supplementary Table S1).

### Statistical analysis

Factorial experiment based on completely randomized design was applied in this experiment with PEG and proline as the first and second experimental factors, respectively. The data were subjected to analysis of variance (ANOVA) and mean comparison based on least significant method (LSD) with *p* = 0.05 using package agricolae in RStudio 1.4.1 software (R core v. 4.0.5). After obtaining the significant letters based on mean comparison, the standard errors of mean (SE) were then calculated for each treatment. Correlation plot according to Pearson linear method with 0.05 significant probability was carried out by ‘ggcorrplot’ library. In addition, heatmap according to Euclidian distance Ward method^22^ using ‘pheatmap’ and ‘gplot2’ library, principal component analysis (PCA) and its and biplot representation based on paired correlation by ‘factoextra’ library, and canonical correlation analysis via ‘CCA’ library were performed.

## Results

### Essential oils, biochemical and growth traits

Table 1 shows multiple mean comparison for measured features containing essential oils contents and biochemical traits. Under all levels of PEG, higher concentration of proline up to 15 µm decreased SOD activity, while application 20 µm increased its activity. On the other side, higher PEG concentration dramatically increased the activity of SOD. Highest SOD activity was recorded for highest water shortage level (20% PEG) and no application of proline. Under 10% PEG, proline showed its highest contents in comparison with other PEG levels. Under no water shortage condition application of 10 µm proline caused the plant to increase content of proline. However, the content of free proline in the surahalala showed increasing pattern in response to higher concentration of proline under both moderate (10% PEG) and severe (20% PEG). Mean comparison of H_2_O_2_ indicated that the higher the content of PEG application in medium, the higher the content of H_2_O_2_ in surahalala plants. On the contrary, increasing the content of proline lead to lower content of H_2_O_2_, except in 20 µm proline level that showed H_2_O_2_ content almost equal to no proline application. Under all water shortage levels, from control to 15 µm application of proline increased the SFW, while higher proline content decreased SFW. Quite the contrary, the sever water deficit conditions lower growth of surahalala plants and lower SFW. RFW showed no specific pattern response to application of proline under no PEG application, but under 10% PEG it showed a growing pattern in response to proline levels. Application of 10% PEG induced the highest RFW in surahalala, while 20% PEG application significantly decreased the root growth comparing with 10% PEG. The RFW under both 10% and 20% PEG application was recorded for 15 µm proline application.

**Table 1:** Mean comparison of PEG by proline levels for the content of essential oils and other measured features in surahalala plants.

PEG	Proline (µm)	SOD	PRO	H_2_O_2_	SFW	RFW	
0%	0	2 ± 0.095h	0.25 ± 0.005h	8.97 ± 0.209ij	16.3 ± 0.38c	1.6 ± 0.032de	
	5	2.6 ± 0.026f	0.55 ± 0.014f	8.18 ± 0.228ij	18.76 ± 0.144b	1.93 ± 0.046b	
	10	1.45 ± 0.019i	0.71 ± 0.013c	8.2 ± 0.137ij	21.41 ± 0.135a	1.4 ± 0.016f	
	15	1.96 ± 0.033h	0.49 ± 0.011f	7.92 ± 0.136j	18.32 ± 0.438b	1.47 ± 0.039ef	
	20	2.9 ± 0.031e	0.42 ± 0.008g	9.21 ± 0.37hi	9.96 ± 0.136d	1.92 ± 0.034b	
10%	0	3.55 ± 0.035d	0.7 ± 0.013cd	13.65 ± 0.307e	6.19 ± 0.149f	2.32 ± 0.027a	
	5	2.46 ± 0.055fg	0.41 ± 0.014g	12.56 ± 0.153ef	5.81 ± 0.5f	2.3 ± 0.025a	
	10	2.35 ± 0.027fg	0.91 ± 0.018a	11.43 ± 0.191fg	7.93 ± 0.185e	1.76 ± 0.035c	
	15	2.29 ± 0.03g	0.85 ± 0.017ab	10.39 ± 0.295gh	10.47 ± 0.103d	1.68 ± 0.028cd	
	20	3.34 ± 0.035d	0.9 ± 0.01a	19.7 ± 0.132b	6.99 ± 0.427ef	0.63 ± 0.029j	
20%	0	7.11 ± 0.033a	0.63 ± 0.017e	22.23 ± 0.173a	5.79 ± 0.244f	0.8 ± 0.046i	
	5	6.31 ± 0.04b	0.63 ± 0.017de	18.67 ± 0.311bcd	4.4 ± 0.232gh	1.33 ± 0.02fg	
	10	4.13 ± 0.039c	0.87 ± 0.008ab	18.39 ± 0.324cd	6.4 ± 0.119f	1.24 ± 0.038g	
	15	2.98 ± 0.045e	0.83 ± 0.018b	17.72 ± 0.15d	5.12 ± 0.256fg	1 ± 0.013h	
	20	6.44 ± 0.086b	0.63 ± 0.013e	19.45 ± 0.328bc	3.25 ± 0.076h	0.71 ± 0.014ij	

PEG	Proline (µm)	1,8-Cineole	Carvone	α-Pinene	Thymol	Estragole	β-Citronellol
0%	0	0.12 ± 0.002h	0.13 ± 0.002i	0.09 ± 0.002g	0.08 ± 0.001h	0.7 ± 0.016ef	0.18 ± 0.004i
	5	0.59 ± 0.02f	0.43 ± 0.025h	0.09 ± 0.002g	1.09 ± 0.033g	0.39 ± 0.018g	0.17 ± 0.02i
	10	0.64 ± 0.011ef	2.63 ± 0.024b	0.09 ± 0.004g	3.02 ± 0.016d	0.24 ± 0.067h	0.3 ± 0.013i
	15	0.84 ± 0.01d	2.33 ± 0.006c	0.03 ± 0.002i	4.16 ± 0.018c	0 ± 0.009j	0.5 ± 0.003h
	20	0.11 ± 0.002h	0.09 ± 0.02ij	0.1 ± 0.002fg	0.78 ± 0.054g	0.01 ± 0.021j	0.3 ± 0.032i
10%	0	0.96 ± 0.011cd	1.87 ± 0.009d	0.11 ± 0.004ef	1.98 ± 0.014e	0.79 ± 0.027de	0.99 ± 0.011f
	5	1.21 ± 0.007b	1.46 ± 0.035e	0.09 ± 0.002g	3.21 ± 0.05d	0.9 ± 0.006cd	1.6 ± 0.006d
	10	1.15 ± 0.019bc	2.74 ± 0.037a	0.08 ± 0.002h	4.13 ± 0.069c	0.61 ± 0.014f	2.73 ± 0.055b
	15	3.25 ± 0.018a	1.52 ± 0.008e	0.12 ± 0.003e	9.22 ± 0.055a	0.44 ± 0.023g	4.17 ± 0.029a
	20	0.02 ± 0.014i	0 ± 0.031j	0.11 ± 0.001ef	7.1 ± 0.069b	0.06 ± 0.002ij	2 ± 0.01c
20%	0	0.69 ± 0.054e	1.8 ± 0.02d	0.18 ± 0.002b	0.96 ± 0.154g	2.85 ± 0.01a	0.67 ± 0.083g
	5	0.45 ± 0.008g	0.67 ± 0.001fg	0.19 ± 0.003a	0.83 ± 0.028g	1.16 ± 0.003b	0.57 ± 0.033gh
	10	1.07 ± 0.002c	0.57 ± 0.001g	0.16 ± 0.002c	3.29 ± 0.013d	0.97 ± 0.002c	1.43 ± 0.006e
	15	0.5 ± 0.003g	0.01 ± 0.002j	0.14 ± 0.002d	1.66 ± 0.118f	0.15 ± 0.001hi	1.67 ± 0.04d
	20	0.01 ± 0.001i	0.68 ± 0.009f	0.17 ± 0.003bc	0.8 ± 0.013g	0.67 ± 0.016f	0.83 ± 0.017f

Mean ± standard error of mean (based on 8 glass containers as replications).

In each column, means with the same letter(s) are not significantly different (LSD 5%).

**Figure 1:**
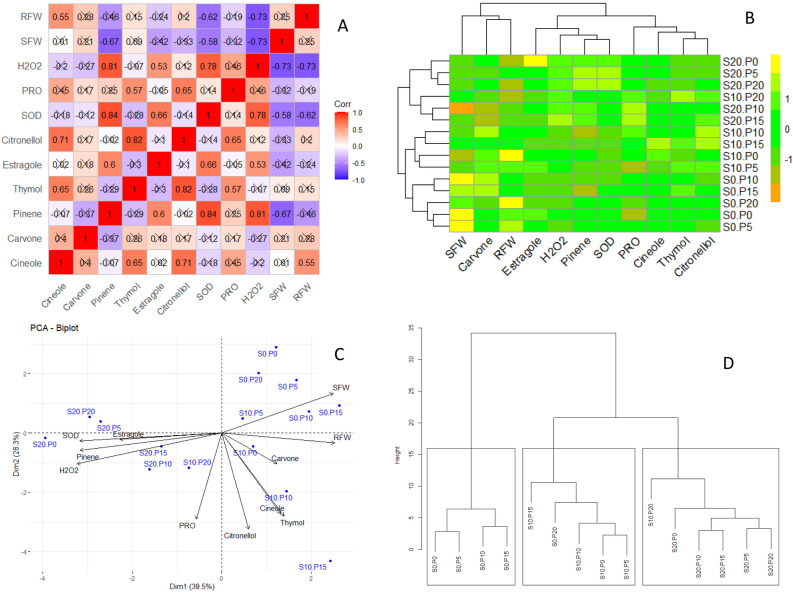
Correlation plot (**A**), heatmap (**B**), biplot (**C**), and clustering (**D**) of the essential oils contents and measured features in surahalala plants under PEG and Proline treatments. The analyses have been conducted on the average of the 8 replications in the experiment. S0_p0: 0% PEG and 0 µm proline; s0_p5: 0% PEG and µm proline; s0_p10: 0% PEG and 10 µm proline; s0_p15: 0% PEG and 15 µm proline; s0_p20: 0% PEG and 20 µm proline; s10_p0: 10% PEG and 0 µm proline; s10_p5: 10% PEG and 5 µm proline; s10_p10: 10% PEG and 10 µm proline; s10_p15: 10% PEG and 15 µm proline; s10_p20: 10% PEG and 20 µm proline; s20_p0: 20% PEG and 0 µm proline; s20_p10: 20% PEG and 10 µm proline; s20_p15: 20% PEG and 15 µm proline; s20_p20: 20% PEG and 20 µm proline; SOD: superoxide dismutase activity; PRO: proline content; H2O2: hydrogen peroxide; SFW: shoot fresh weight; RFW: root fresh weight.

Applying the data mining methods and multiple mean comparison indicated that the contents of all six measured essential oils, generally, grew up in response to PEG application. The α-pinene and estragole showed their highest content under 20% application of PEG; however, the contents of 1,8-cineole, carvone, thymol, and β-citronellol were the highest under 10% application of PEG. Also, α-pinene and estragole showed reduction patterns in response to higher proline levels, while for other essential oils, 10 and 15 µm proline application brought about the highest contents. On the other hand, estragole and α-pinene essential oils contents showed positive and significant correlation with each other while they had no significant correlation with 1,8-cineole, Thymol, and β-citronellol (Fig. 1A). 1,8-cineole, Thymol, and β-citronellol showed positive and significant correlations with one another and their highest correlation was between Thymol and β-citronellol (0.82). Carvone showed negative correlations with estragole and α-pinene and its correlation with other three essential oils content was not significant. Cluster analysis of the essential oils showed the similar pattern in which estragole and α-pinene were placed into one cluster, carvone was contained in one cluster and Thymol, β-citronellol, and 1,8-cineole were set into another separate cluster (Fig. 1B). Biplot showed that Carvone along with Thymol, β-citronellol, and 1,8-cineole were placed in the same quarter which contained 15%, 10%, and no application of proline under 10% PEG treatment (Fig. 1C).

Pearson correlation indicated that SOD, proline content, and H_2_O_2_ had positive significant correlations with one another while they presented negative correlations with root and shoot weight of surahalala (Fig. 1A). Additionally, SFW and RFW were grouped in the cluster of carvone (Fig. 1B). The cluster of estragole and α-pinene contained SOD and H_2_O_2_ content. According to cluster analysis of treatments (Fig. 1D) and the biplot, except 20 µm proline, all other levels of proline treatments under no PEG application were grouped together with high similarity. Application of 20 µm proline under no PEG application was contained in the cluster of 10% PEG application.

### Gene expression profiling

**Figure 2:**
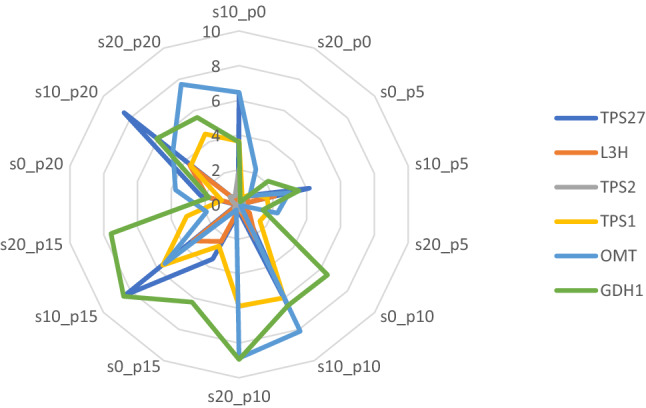
Radar graph for comparing relative expression levels of the used genes under different treatment levels of PEG and proline in surahalala plants. The analyses have been conducted on the average of the 8 replications in the experiment. S0_p0: 0% PEG and 0 µm proline; s0_p5: 0% PEG and µm proline; s0_p10: 0% PEG and 10 µm proline; s0_p15: 0% PEG and 15 µm proline; s0_p20: 0% PEG and 20 µm proline; s10_p0: 10% PEG and 0 µm proline; s10_p5: 10% PEG and 5 µm proline; s10_p10: 10% PEG and 10 µm proline; s10_p15: 10% PEG and 15 µm proline; s10_p20: 10% PEG and 20 µm proline; s20_p0: 20% PEG and 0 µm proline; s20_p10: 20% PEG and 10 µm proline; s20_p15: 20% PEG and 15 µm proline; s20_p20: 20% PEG and 20 µm proline.

Expression profiling of six genes involved in essential oils and secondary components in surahalala plant grown under in vitro condition were assessed and their expression levels were standardized according to two different reference genes, EF1 and actin, which their mean comparison in each treatment level is represented in Supplementary Tables S2 and S3. Since the correlation between the results related to both references genes were high (over 0.85), their mean profiling has used as final expression levels and the mean comparisons of the treatments regarding all the six genes are provided in Table 2. The expression of 1,8-cineole, Carvone, Alph_P, and β-citronellol genes in 10% PEG application were higher than other control (no PEG) and 20% PEG; however, 20% PEG application showed expression level for Thymol and estragole (Table 2). Application of different levels of proline under 20% and no PEG application showed caused some small changes in expression profiling of the considered genes. On other hand, these genes have strongly responded to proline application under 10% PEG application and their expression went through much changes. Under 10% PEG treatment, 10 and 15 µm proline caused higher expression rate than other proline levels. Figure 2 shows the expression rate of all considered genes versus the treatment levels. Generally, the expression level of 1,8-cineole and β-citronellol genes were higher than other genes particularly under 10 and 15 µm proline levels along with 10 and 20% PEG applications.

**Table 2:** Mean comparison of PEG by proline levels for expression rate of the selected genes in surahalala plants.

PEG	Proline	TPS27	L3H	TPS2	TPS1	OMT	GDH1
0	0	1 ± 0f	1 ± 0f	1 ± 0b	1 ± 0i	1 ± 0hi	1 ± 0gh
0	5	0.84 ± 0.012f	0.78 ± 0.011fg	0.27 ± 0.006fg	1.02 ± 0.014i	0.8 ± 0.013hi	2.14 ± 0.05fg
0	10	0.39 ± 0.004f	0.77 ± 0.008fg	0.08 ± 0.001i	1.54 ± 0.015h	0.1 ± 0.001i	6.53 ± 0.09e
0	15	3.5 ± 0.053e	2.38 ± 0.025e	0.2 ± 0.003h	2.67 ± 0.034g	0.36 ± 0.004i	6.25 ± 0.13e
0	20	2.27 ± 0.069ef	1.88 ± 0.034e	0.31 ± 0.005ef	1.02 ± 0.017i	3.76 ± 0.071f	1.78 ± 0.03gh
10	0	6.4 ± 0.085d	4.81 ± 0.049d	2.07 ± 0.028a	3.63 ± 0.038f	6.45 ± 0.064e	3.59 ± 0.059f
10	5	4.16 ± 0.089de	2.11 ± 0.041e	0.28 ± 0.004fg	1.66 ± 0.019h	2.95 ± 0.054fg	3.54 ± 0.047f
10	10	30.39 ± 0.583b	18.22 ± 0.344a	0.54 ± 0.013d	10.98 ± 0.203c	8.13 ± 0.168d	46.49 ± 1.211a
10	15	94.39 ± 1.73a	13.38 ± 0.203b	0.35 ± 0.005e	11.63 ± 0.12b	5.48 ± 0.116e	27.51 ± 0.329b
10	20	21.48 ± 0.427c	10.64 ± 0.209c	0.68 ± 0.018c	3.56 ± 0.067f	34.88 ± 0.707b	6.08 ± 0.181e
20	0	0.1 ± 0.002f	0.01 ± 0.001h	0.11 ± 0.001i	0.42 ± 0.005j	2.24 ± 0.034fgh	0.16 ± 0.002h
20	5	0.1 ± 0.003f	0.01 ± 0.002h	0.09 ± 0.001i	1.76 ± 0.017h	2.27 ± 0.036fgh	1.42 ± 0.02gh
20	10	0.42 ± 0.006f	0.25 ± 0.003gh	0.24 ± 0.003gh	9.87 ± 0.108d	28.87 ± 0.514c	15.94 ± 0.202c
20	15	0.13 ± 0.001f	0.07 ± 0.001h	0.11 ± 0.003i	14.21 ± 0.247a	1.92 ± 0.021gh	12.57 ± 0.354d
20	20	0.15 ± 0.003f	0.17 ± 0.002h	0.53 ± 0.006d	8.5 ± 0.083e	62.68 ± 0.797a	5.56 ± 0.073e

Mean ± Standard error (based on 3 glass containers as replications).

In each column, means with the same letter(s) are not significantly different (LSD 5%).

**Figure 3:**
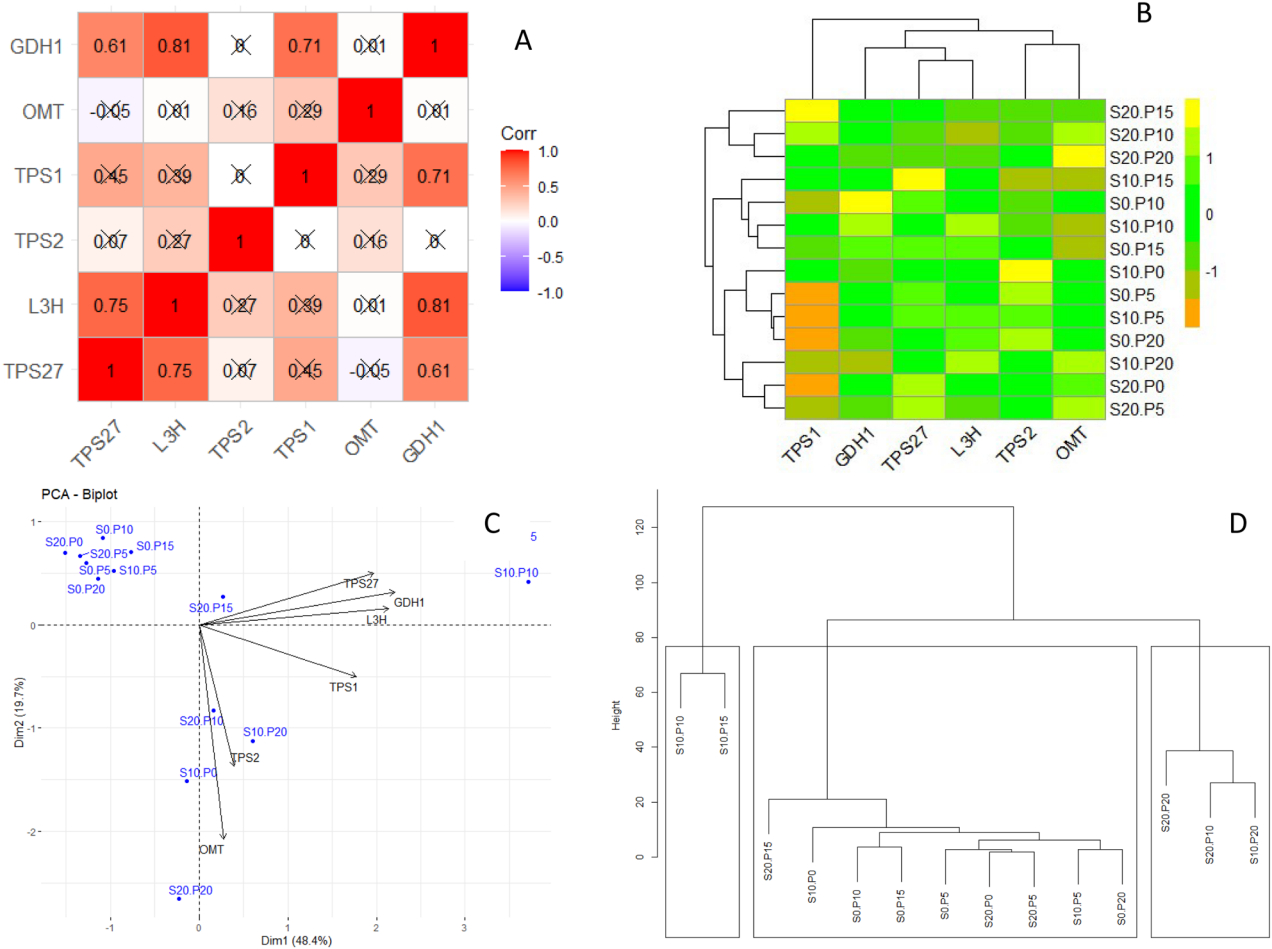
Correlation plot (**A**), heatmap (**B**), biplot (**C**), and clustering (**D**) of the expression profiling of essential oils genes in surahalala plant under PEG and Proline treatments. The analyses have been conducted on the average of the 8 replications in the experiment. S0_p0: 0% PEG and 0 µm proline; s0_p5: 0% PEG and µm proline; s0_p10: 0% PEG and 10 µm proline; s0_p15: 0% PEG and 15 µm proline; s0_p20: 0% PEG and 20 µm proline; s10_p0: 10% PEG and 0 µm proline; s10_p5: 10% PEG and 5 µm proline; s10_p10: 10% PEG and 10 µm proline; s10_p15: 10% PEG and 15 µm proline; s10_p20: 10% PEG and 20 µm proline; s20_p0: 20% PEG and 0 µm proline; s20_p10: 20% PEG and 10 µm proline; s20_p15: 20% PEG and 15 µm proline; s20_p20: 20% PEG and 20 µm proline.

Correlation coefficient analysis indicated positive and significant association of Carvone, β-citronellol, and 1,8-cineole with one another (Fig. 3A). Accordingly, clustering of the considered genes placed Carvone, β-citronellol, and 1,8-cineole into one cluster, α-pinene and estragole in a separate cluster, and Thymol in another cluster (Fig. 3B). These results were clearly verified by biplot where genes in the same cluster were placed near to each other (Fig. 3C). According to clustering results of treatments (Fig. 3D), all treatment levels were incorporated with three separate clusters where 10 and 15 µm under 10% PEG treatment were closely placed in the same cluster. Also, the clustering results of the treatments were corroborated by biplot results.

**Figure 4:**
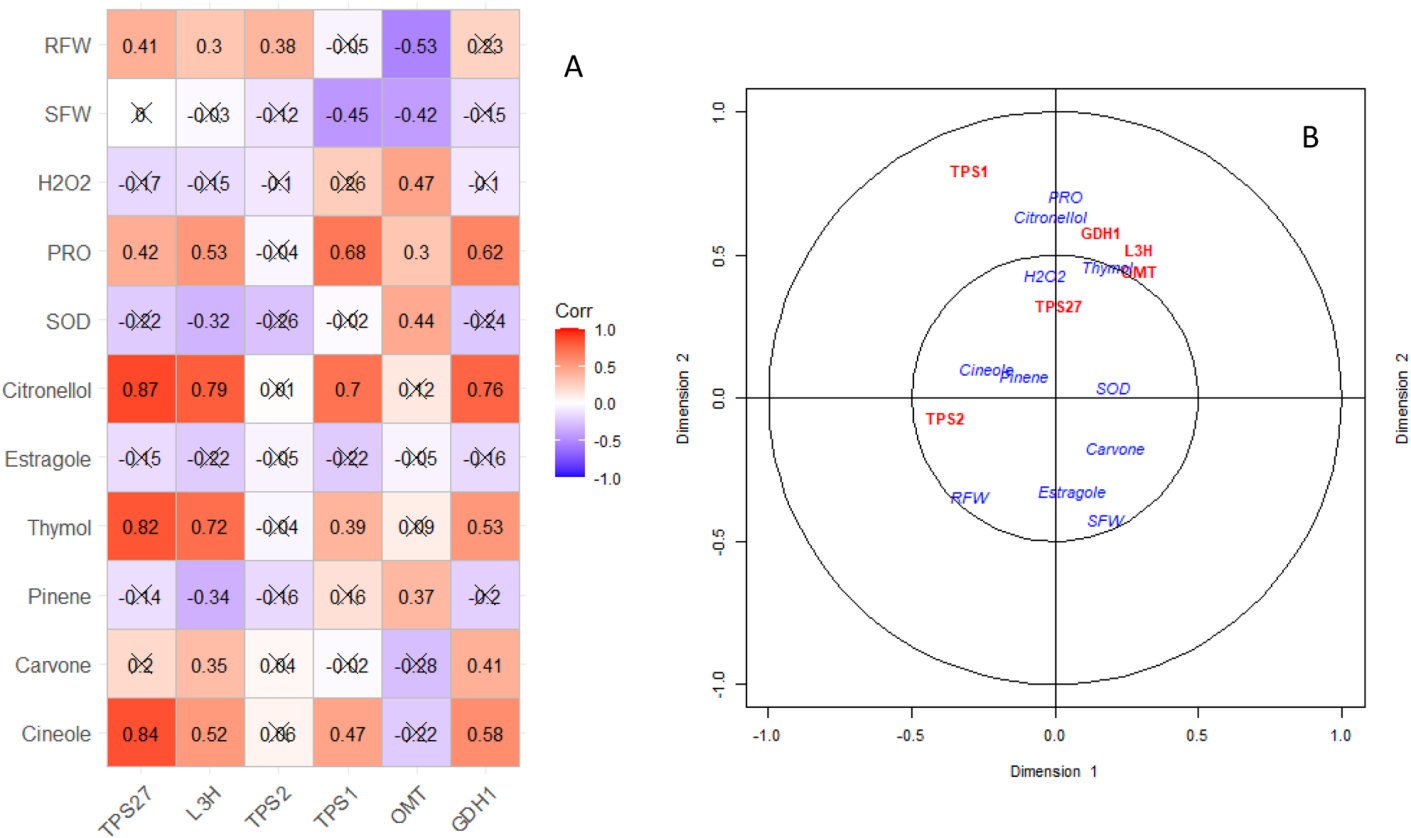
Correlation plot (**A**) and canonical correlation biplot (**B**) for relative expression of the selected gene versus essential oils content and other measured features in surahalala plants. The analyses have been conducted on the average of the 8 replications in the experiment. SOD: superoxide dismutase activity, PRO: proline content, H_2_O_2_: hydrogen peroxide, SFW: shoot fresh weight, RFW: root fresh weight.

Cross correlation between expression of the considered genes versus the biochemical and growth-related features is presented in Fig. 4A. Accordingly, the expression rate of 1,8-cineole gene had the highest correlation (0.84) with its essential oil component, 1,8-cineole contents. The expression level of α-pinene was negatively correlated with α-pinene content. In order to summarize these cross associations canonical correlation for expression profiling as one set and other measured features as another set was carried out. The results showed that the first four canonical coefficient (CC) out of six showed significant explanation of cross relationship of the two sets (Supplementary Table S4). The members of the two sets were portrayed as 2D-plot based on the first and second CC (CC1 and CC2) in which proline content showed a close proximity with expression rate of Thymol, β-citronellol, Carvone, and estragole along with the content of β-citronellol and H_2_O_2_ (Fig. 4B). The activity of superoxide dismutase was placed near to the contents of Carvone, α-pinene, and 1,8-cineole essential oils. Among the expression profiling of the six genes, α-pinene expression rate was closest to shoot and root weights of surahalala.

## Discussion

Up to the best of our knowledge, there is no study on the effect of environmental stresses on surahalala plants. However, according to previous studies^23–25^, essential oil contents and the expression profiling of their related genes along with the growth of aromatic plants are significantly affected by altering the environmental conditions. Chrysargyris et al.^26^ reported that water deficit is one the major environmental factors changing the synthesis and production of secondary metabolite in medicinal plants. Moreover the such stress condition can cause lower plant growth and matter production in significant amounts. On the other hand, proline has reported to be an effective free amino acid in response to environmental stresses where it can play as osmoregulatory elements in plants. In most of cases application exogenous proline led to higher tolerant in plants under water deficit stress. However, its application has sowed different effects on the contents of secondary metabolites and the expressional levels of their related genes^27–32^.

In order to quantify the effect of PEG application as a simulator of water deficit condition and the effect of proline in such situation, SOD activity and H_2_O_2_ content were measured. The results according to higher H_2_O_2_ content and SOD activity under higher PEG levels showed that PEG application was able to properly simulate the stress condition. In addition, the content of free proline increased in response to PEG application in surahalala plants. Though, PEG led to significant decrease in plant growth by affecting the shoot and root weights. Moreover, the results of proline application on SOD activity and H_2_O_2_, on one hand, and on fresh shott and root weights of the surahalala plants, on the other hand, indicated that higher concentration of proline than 15 µm might causes stress-like condition in this plant. The application of proline up to 15 µm showed mainly decrease in the SOD activity and the content of H_2_O_2_ along with higher shoot and root weights in comparison with the control, under all PEG levels. The content of proline, however, was continuously increased in response to higher level of exogenous application of proline. These results were verified by different advanced data mining methods in which the associations of SOD and H_2_O_2_ with root and shoot weights were highly negative. In most previous studies application of proline resulted in higher dry mater production and the tolerance of the plants^33^. Although, in our study was verified that higher contents of proline than a critical point might lead to negative effect on the plants. In concordance with our study, different authors showed that proline regulation application was able to decrease the negative effects of environmental stress on *Trifolium repens* L.^30^, tobacco^32^, *Glycyrrhiza uralensis*^27^, and chickpea^28^ mostly via decreasing the content of H_2_O_2_ and malondialdehyde and also increasing the activity of enzymatic antioxidants. Also, Zali and Ehsanzadeh^33^ and^34^ Showed that proline was capable of increasing the growth of fennel And *Phaseolus vulgaris* under water deficit conditions and nutrient deficiency.

Measuring secondary metabolites in the current study indicated that PEG application has positive effect on the contents of most essential oils. On the other hand, in all metabolite such as 1,8-cineole, Carvone, Thymol, and β-citronellol the highest PEG level (20%) did not show highest content, instead it showed lower contents in compare with 10% level of PEG. This result indicates that environmental stresses might play a role in activating some genes that are involved in both essential oils synthesis pathways and stress responsive genes. Nonetheless, the complex polyploidy levels in line with lack of genomic and transcriptomic information related to the majority of aromatic plants have limited further study even on model plants such as mint species^35^. In some studies^23,36,37^, associations between genes that are involved in ABA, jasmonic acid and gibberellin responsive pathways which are enhanced under specific stresses and the metabolite gene network have been reported.

In line with our results, Akula and Ravishankar^38^ and Aftab^39^ reported higher secondary metabolites in response to environmental stresses, but there is no study related to the stress effect on metabolite contents of surahalala. Meanwhile, there were positive association between 1,8-cineole, Carvone, Thymol, and β-citronellol contents with proline content in surahalala and they showed low Euclidian distances from one another as well as placing in similar clusters. These essential oils were increased by increasing the level of proline up to 15 µm and then decreased dramatically. The α-pinene and estragole contents showed negative response to proline application. Since 1,8-cineole, carvone, thymol, and β-citronellol levels were increased in response to 10% PEG and up to 15 µm proline in surahalala plants, it seems that low water deficit condition and moderate application of proline may a proper combination of treatment leading to increase their concentrations per dry matter unit in this plant. On the other hand, α-pinene and estragole showed negative associations with proline, but their contents were generally equal to, or in some cases higher than, the control proline treatment. Therefore, the suggested treatment combination of low water deficit level and moderate application of proline would not have significant negative impacts on these compounds.

As a class of secondary metabolites, monoterpenes are mainly identified in fungi, but different contents of them have also been reported in some medicinal plants such as eucalyptus^40^ pepper mint, and recently in surahalala^2^. These compounds showed variety functions in plants and are involved in some basic and specialized metabolisms. Some of these compounds play a role in odor synthesizing for attracting the pollinators. There are reports related to their operations in defense mechanisms against herbivores and plant pathogens by synthesizing some toxic compounds. There are some studies indicating that these compounds are involved in sinical transduction under various stresses after the starting damage to the plant cell^2^. In the current study, H_2_O_2_ as signaling molecule showed significant and positive associations with α-pinene and estragole based on the results of data mining which are concordance the previous studies^33,41^. Other reports regarding the importance of monoterpenes are related to their impacts on symbiosis relationship of their plants by accumulating in their roots and rhizomes^42,43^. In addition to the importance of monoterpenes on the plants, different effects of these compounds on human diseases have been reported.

In aromatic and pharmaceutical plants, the content of essential oils and terpenoid compounds are important substances with high economic values. The major contents of surahalala essential oils, according to Mozafari et al.^2^, are consisted of monoterpenes. 1,8-cineole is a commercially significant monoterpene compound that has pharmaceutical applications and is considered as a potential biofuel. According to the literatures, this compound has anti-inflammatory impacts and Linghu et al.^44^ reported that it was able to subside the inflammatory effect of human umbilical vein endothelial cells (HUVECs) disease phenotype^45^. Similarly, carvone is known to have anticarcinogenic properties in human tissues. Carvone is also able to synthesis attractive odors and flavors applicable in aromatherapy and food products^46^. In our study carvone content showed high Euclidian distances from SOD activity and H_2_O_2_ indicating revers associations between these compounds in some conditions. In line with these results, Huchelmann et al.^47^ described that carvone is able to hinder the production of a stress-induced compounds and metabolites in tabacum leaf. In addition to 1,8-cineole, pinene has anti-inflammatory and also antimicrobial activities. Surahalala plants under the water deficit and proline treatments showed to have thymol in their shoot which is able to be used as an effective antimicrobial compound. Having antiseptic effects caused thymol to be used as ingredient in mouthwash and toothpaste products^41^. Another monoterpene that was distinguished in surahalala plants is estragole which can act as an agent for attracting the pollinators, play a role in defense mechanism against pathogens and herbivores, and be applied in food products and spices as flavoring compounds^48^. Similarly, β-citronellol is used in perfume factories and can act in pollinators attractions along with repellent effects on some organisms mainly mosquito^49^.

As it verified by the results of this study and mentioned in the results of some other studies^6,11,15,17^, different the environmental conditions lead to change in the contents of secondary metabolites. The contents and biosynthesis of the monoterpenes are normally regulated by similar or different molecular and genomic pathways which are most commonly functioning as the connected gene networks. Altering environmental conditions or treating plants with various compounds may directly trigger or repress some genes in monoterpenes genomic pathways, or the genes that are responsible for transcribing their sequences into RNAs. The levels of changes under altered conditions could be quantified by expression rates of related genes and considering the transcriptomic patterns of the plants’ tissues. In this study, all considered monoterpenes were affected by the stress condition and proline treatments. Hence, assessing the expression rates of the genes involved in their biosynthesis can help us to find the origin of the changes in such metabolites and lead the way to future genetic engineering toward upregulate or downregulate their expression. The monoterpenes that are considered in this study are the product of enzymatic activities transforming specific precursors.

According to Chen et al.^35^, terpene synthase (TPS) gene family is largely involved in terpenes biosynthesis of *Mentha longifolia*. In their study, TPS family was divided into six subfamilies sharing a great number of mostly identical and some similar motifs. After considering these motifs in all available data bases of the aromatic plants we found over 90% similarity among these motifs indicating that these subfamilies are most likely available in surahalala with similar sequences and tasks. However, sequence analyses of complete cds of different TPSs within and between different species by Huchelmann et al.^47^ indicated that the similarity of these genes within each species genome is significantly higher than the similarity between genomes of different species.

Almost all of the precursors of different monoterpenes are product of similar pathways in which TPS genes act in nearly end of these pathways. In these pathways, isopentenyl diphosphate (IPP) and dimethylallyl diphosphate (DMAPP) are synthesized at the initial points. IPP and DMAPP are direct products of two other pathways consist of mevalonate pathway (MVA) in the cytosol and phosphate pathway (MEP) in the chloroplast. Generally, IPP and DMAPP are directly transferred into geranylgeranyl pyrophosphate (GGPP), farnesyl diphosphate (FPP), and geranyl diphosphate (GPP) by catalyzing activities of prenyltransferases. After that, different TPS genes catalyze these compounds into the precursors of monoterpenes and other secondary metabolites. The produced precursors are then modified by enzymatic activities transcribed from different enzymatic genes^35^.

The content of 1,8-cineole in this study was higher than its content in other plants, and the proline treatments specially under water deficit condition sufficiently increased the levels of this monoterpene. Huchelmann et al.^47^ attributed the low level of 1,8-Cineole in *nicotiana tabacum* to rapid conversion of GPP precursor into other monoterpenes other than 1,8-Cineole. Therefore, proline treatment water deficit condition most likely trigger the expression of some genes involved in either higher production of 1,8-Cineole precursor or more rapid conversion of the precursor GPP into this monoterpene in surahalala plants. Other than treated surahalala plants, control plants showed slightly higher 1,8-cineole content than other plants. In study of Chen et al.^50^, 1,8-Cineole has shown negative effects on germination and significantly reduced its percent in *Arabidopsis*; consequently, the problems with direct germination of surahalala plants may be the results of higher 1,8-cineole content in this plant which led us to produce it under in vitro condition. On the other hand, one of genes involved in production of 1,8-cineole precursor is TPS27, which its relative expression rate in surahalala plant under moderate water deficit and slight concentration of proline was significantly higher than control conditions and high concentrations of PEG (20%) and proline (20 µm).

The expression rate of L3H gene was significantly increased by applying PEG and proline treatments in surahalala which its highest rate was achieved by 10 or 15 µm proline under 10% PEG. The expression rate of this gene showed positive and significant correlation with carvone content in surahalala and based on the data mining results, the Euclidian distance of this monoterpene and L3H expression rate was the significantly lower than other gene expression profiling. Carvone results from MEP pathway that produces geranyl diphosphate (GDP) and it showed negative regulatory effects on MVM pathway resulted in negative association and high distance between this monoterpene and other assessed monoterpene in this study, for example estragole, that are produced by MVM pathway. After biosynthesizing of GDP, limonene synthase (LS) enzyme converts it to limonene by separating the diphosphate group. Next, hydroxylation of limonene (C6 position) by L3H and L6H by co-factoring of NADPH transforms it into carveol and finally into carvone by dehydrogenases mechanism^51^. One of the reasons for increasing the content of carvone under water deficit condition in the current study may be the production of signaling molecule such O^-^ and OH^-^ that are capable of dehydrogenating different compounds. On the other hand, proline might get involved directly in L3H production pathway or indirectly help NADPH cofactor in carvone pathway, lead to higher expression profiling of L3H by both ways. In the study of Xie et al.^49^ higher gibberellin content, which is affected by stress conditions, showed positive interaction with carvone content.

The anti-inflammatory and antimicrobial of α-pinene have verified by different studies. According to Wu et al.^52^, α-pinene is a specific product from aromatic plants which the most of other organisms are not able to produce it. α-pinene is directly synthesized by the activity of pinene synthase (PS) enzyme from GGPP. In the current study, the expression rate of TPS2 gene was increased in response to application 10% PEG, while it decreases by application 20% PEG. This result indicates that moderate stress on surahalala plant can lead to higher conversion of GGPP into this monoterpene. The results of data mining verified these results and showed that the expression rate of TPS2 and also the content of α-pinene were positively correlated and they had low Euclidian distance from those treatments that contained 10% PEG in two-dimensional representation of canonical correlation. Under no stress and sever stress conditions expression rate of TPS2 showed no regular pattern in response to proline treatments, while its rate was the highest under moderate stress level (10%PEG) with no proline application. Therefore, proline treatment has a negative regulatory effect on the expression rate of TPS2. To the best of our knowledge, there is no study related to the effect of proline treatment on expression rate of TPS2 gene or the content of α-pinene. Wu et al.^52^ showed that in addition to enzymatic activity of TPS2, fusion of the pinene synthase (PS) with heterologous geranyl diphosphate synthase (GPPS), they called GPPS-PS significantly increased the content of α-pinene and β-pinene.

In the previous study by Hoseiny et al.^2^, thymol was detected in surahalala shoots. In the current study, the content of thymol was increased as the results of proline treatments and PEG application, however, the best combination for achieving higher thymol content was 15 µm proline level applied under 10% PEG as moderate water deficit condition. One pathway of biosynthesis of thymol is through conversion of GPP to neryl pyrophosphate (NPP) which in turn it transforms to γ-terpinene and then into p-cymene. The final product of this pathway is either carvacrol or thymol. In surahalala both of these monoterpenes were detected by gas chromatography method used in this study; though, the content of thymol was higher in almost all samples. Majdi et al.^53^ described TPS1 as a significant agent in thymol biosynthesis. The expression profiling of this gene (TPS1) showed similar pattern to the content of thymol in response to the treatments. The main product of TPS1 activity in metabolite biosynthesis pathway is to γ-terpinene, one of the precursors of thymol. Canonical correlation and correlation plot showed that TPS1 gene expression in addition to low two-dimensional distance and high correlation with thymol, it showed a close relationship with β-Citronellol content as well. Additionally, the relative expression of TPS1 showed significant association with 1,8-cineole content based on the results from heatmap analysis. This means that TPS1 is probably involved in other metabolite biosynthesis pathways in surahalala and it likely is not a pathway specific enzyme.

The availability of estragole metabolite in surahalala was first reported by Hoseiny et al.^2^. The importance of this metabolite in surahalala is related to its role as a defense compounds against different microorganisms and herbivores, in addition to pollinator attractant ^48^. Estragole is a subset of phenylpropenes group that also contain isoeugenol, eugenol, and transanethole (isoestragole). A part of flavoring properties of some plant species such as banana (*Musa sapientum*)^54^, melon (*Cucumis melo*)^55^, tomato (*Solanum lycopersicum*)^56^, and strawberry (*Fragaria vesca*)^57^ is resulting from the phenylpropenes group available in these fruit in their sequestered glycosides or as free volatiles forms. Estragole is normally biosynthesized via IPP pathway which its precursor are the compounds of para-hydroxy group such as coniferyl acetate^41^ and p-allylphenol^58^. O-methyltransferases (OMTs) is an important enzyme act in transforming para-hydroxy compounds to estragole by using a methyl donor (S-adenosylmethionine; SAM)^48^. The content of estragole in surahalala plants were significantly increased in response to higher water deficit condition and higher proline treatments. Similarly, the expression profiling of OMT genes showed positive response to these treatments. Unlike other considered metabolites in this study, the highest content of estragole in surahalala plants was obtained in highest proline level (20 µm) under highest PEG concentration (20%). The effectiveness of these treatments comes from their impact on methyl productions pathways because some methylated compounds could be used in signal transduction activity in response to stress conditions. As it was mentioned earlier, the highest level of proline in surahalala plant is probably causes nutrient stress or make water deficit severer leading to activating some networks in stress signaling pathways that take advantage of methylated compounds.

β-Citronellol is another metabolite that was detected in surahalala and its availability in this plant was also reported previously by Hoseiny et al. ^2^. This metabolite significantly increases the importance of using surahalala extracts in industrial products as the result of its application in insect (specially mosquito) repellents and perfumes^59^. β-Citronellol content and the expression rate of geraniol dehydrogenase GDH3 showed significant and positive correlation (0.73) resulting from their similar patterns in response to PEG and proline application in this study. Base on the clustering results of metabolites and the differential expressions of considered genes in this study obtained from heatmap method, GDH3 expression rate and β-Citronellol showed the closest relationship with 10 and 15 µm proline and 10% PEG treatments. GDH is one the significant enzymes act in biosynthesis of geraniol and citronellol by dehydrogenizes the GPP precursors. Moreover, GDH3 expression rate showed low Euclidian distances in two-dimensional plots of biplot and canonical correlation from TPS1 and L3H expression rates and the thymol content in surahalala.

## Conclusion

The results of this study clearly verified that *Hymenocrater longiflorus* as a hardly known plant species is capable of being used and developed as an important pharmaceutical plant. Considering the impacts of water stress by PEG application and proline treatment showed that water deficit increases oxidants levels while decreases fresh weight of surahalala tissues; whereas, application of proline up to 15 µm was able to relatively compensate the negative effect of water deficit. The results also indicated that high proline level (over 20 µm) can act stress simulator in surahalala plants and have negative effect on its growth. In addition, the best combination of proline and PEG treatment in surahalala plant for achieving highest content its essential oils were 10 µm and 10% levels, respectively. Even though the sequence of different genes in surahalala is unknown, we could saucerful assessed the expressional profiling of TPS27, L3H, TPS2, TPS1, OMT and GDH3 in this plant by considering the sequences of these genes in closely related plant specious such *Salvia officinalis L*, *Hybrid lavandin (L. angustifolia* × *L. latifolia*)*, Origanum vulgare*, *Melissa officinalis*, *Dracocephalum moldavica*, *Majorana hortensis syn. Origanum majorana*, etc., and distinguishing highly similar domains. These genes showed to be actively involved in 1,8-cineole, carvone, α-pinene, thymol, estragole and β-Citronellol synthesis, respectively. In addition, our results indicated that these genes could get involved in other metabolite synthesis under water deficit condition. Additionally, a R package was developed in this study that is able to estimate the relative expressional rate of any considered gene by taking its cycle threshold (Ct) point of internal and target gene.

## Supplementary Information

Supplementary Information 1.

Supplementary Information 2.

Supplementary Information 3.

Supplementary Information 4.

Supplementary Tables.

## Abbreviations

SOD: Superoxide dismutase
POD: Peroxidase
SV: Simvastatin
ABA: Abscisic acid
BAP: Benzyl aminopurine
IBA: Butyric acid
PEG: Polyethylene glycol
Pro: Proline
SFW: Shoot fresh weight
RFW: Root fresh weight
H2O2: Hydrogen peroxide
ANOVA: Analysis of variance
LSD: Least significant method
SE: Standard errors of mean
PCA: Principal component analysis
CCA: Canonical correlation analysis

## References

[1] Ahmadi F, Sadeghi S, Modarresi M, Abiri R, Mikaeli A (2010). Chemical composition, in vitro anti-microbial, antifungal and antioxidant activities of the essential oil and methanolic extract of *Hymenocrater longiflorus* Benth., of Iran. Food Chem. Toxicol..

[2] Hoseiny M, Mozafari AA, Nazari F (2021). The antagonistic relationships between salicylic acid and simvastatin on phyto-biochemical components in *Hymenocrater longiflorus* Benth under in vitro conditions. Ind. Crops Prod..

[3] Shahriari S, Khanahmadi M, Tahvilian R (2013). The study of essential oil of *Hymenocrater longiflorus* Benth growing in Paveh. J. Rep. Pharm..

[4] Ni Z-J (2021). Recent updates on the chemistry, bioactivities, mode of action, and industrial applications of plant essential oils. Trends Food Sci. Technol..

[5] Abdelmajeed NA, Danial EN, Ayad HS (2013). The effect of environmental stress on qualitative and quantitative essential oil of aromatic and medicinal plants. Arch. Sci..

[6] Langroudi ME, Sedaghathoor S, Bidarigh S (2013). Effect of different salinity levels on the composition of rosemary (*Rosmarinus officinalis*) essential oils. AEJAES.

[7] Saed-Moucheshi A (2021). Superoxide dismutase (SOD) as a selection criterion for triticale grain yield under drought stress: A comprehensive study on genomics and expression profiling, bioinformatics, heritability, and phenotypic variability. BMC Plant Biol..

[8] Shakeri E, Mozafari AA, Sohrabi F, Saed-Moucheshi A (2019). Handbook of Plant and Crop Stress.

[9] Saed-Moucheshi A, Pessarakli M, Mikhak A, Ostovar P, Ahamadi-Niaz A (2017). Investigative approaches associated with plausible chemical and biochemical markers for screening wheat genotypes under salinity stress. JPN..

[10] Saed-Moucheshi A, Shekoofa A, Pessarakli M (2014). Reactive oxygen species (ROS) generation and detoxifying in plants. JPN..

[11] Nounjan N, Nghia PT, Theerakulpisut P (2012). Exogenous proline and trehalose promote recovery of rice seedlings from salt-stress and differentially modulate antioxidant enzymes and expression of related genes. J. Plant Physiol..

[12] Cao X (2020). Abscisic acid mediated proline biosynthesis and antioxidant ability in roots of two different rice genotypes under hypoxic stress. BMC Plant Biol..

[13] Li J (2018). OsERF71 confers drought tolerance via modulating ABA signaling and proline biosynthesis. Plant Sci..

[14] Lee B-R (2019). Characterization of salicylic acid-mediated modulation of the drought stress responses: Reactive oxygen species, proline, and redox state in *Brassica napus*. Environ. Exp. Bot..

[15] Poorghadir M, Torkashvand AM, Mirjalili SA, Moradi P (2020). Interactions of amino acids (proline and phenylalanine) and biostimulants (salicylic acid and chitosan) on the growth and essential oil components of savory (*Satureja hortensis* L.). Biocatal. Agric..

[16] Sofy MR, Seleiman MF, Alhammad BA, Alharbi BM, Mohamed HI (2020). Minimizing adverse effects of pb on maize plants by combined treatment with jasmonic, salicylic acids and proline. Agronomy.

[17] Werrie P-Y, Durenne B, Delaplace P, Fauconnier M-L (2020). Phytotoxicity of essential oils: Opportunities and constraints for the development of biopesticides—A review. Foods..

[18] Murashige TC, Skoog F (1962). A revised medium for rapid growth and bioassays with tobacco tissue cultures. Physiol. Plant..

[19] Bates L, Waldren R, Teare I (1973). Rapid determination of free proline for water-stress studies. Plant Soil..

[20] Beers RF, Sizer IW (1952). A spectrophotometric method for measuring the breakdown of hydrogen peroxide by catalase. J. Biol. Chem..

[21] Dhindsa RS, Plumb-Dhindsa P, Thorpe TA (1981). Leaf senescence: Correlated with increased levels of membrane permeability and lipid peroxidation, and decreased levels of superoxide dismutase and catalase. J. Exp. Bot..

[22] Saed-Moucheshi A, Fasihfar E, Hasheminasab H, Rahmani A, Ahmadi A (2013). A review on applied multivariate statistical techniques in agriculture and plant science. Intl J. Agron. Plant Prod..

[23] Pirbalouti AG, Samani MR, Hashemi M, Zeinali H (2014). Salicylic acid affects growth, essential oil and chemical compositions of thyme (*Thymus daenensis* Celak.) under reduced irrigation. Plant Growth Regul..

[24] Çakmakçı R, Mosber G, Milton AH, Alatürk F, Ali B (2020). The effect of auxin and auxin-producing bacteria on the growth, essential oil yield, and composition in medicinal and aromatic plants. Curr. Microbiol..

[25] Morshedloo MR (2017). Effect of prolonged water stress on essential oil content, compositions and gene expression patterns of mono-and sesquiterpene synthesis in two oregano (*Origanum vulgare* L.) subspecies. Plant Physiol. Biochem..

[26] Chrysargyris A, Mikallou M, Petropoulos S, Tzortzakis N (2020). Profiling of essential oils components and polyphenols for their antioxidant activity of medicinal and aromatic plants grown in different environmental conditions. Agronomy.

[27] Cui G (2021). Exogenous silicon relieve drought stress and salt stress of *Glycyrrhiza uralensis* seedlings by regulating proline metabolism and nitrogen assimilation. J. Hortic. Sci. Biotechnol..

[28] Hussain N, Yasmeen A, Yousaf MM (2021). Antioxidant status and their enhancements strategies for water stress tolerance in chickpea. Braz. J. Biol..

[29] Ganguly M, Roychoudhury A, Sengupta DN, Datta SK, Datta K (2020). Independent overexpression of OsRab16A and AtDREB1A exhibit enhanced drought tolerance in transgenic aromatic rice variety Pusa Sugandhi 2. J. Plant Biochem. Biotechnol..

[30] Li Z (2014). Exogenous spermidine improves water stress tolerance of white clover (*Trifolium repens* L.) involved in antioxidant defence, gene expression and proline metabolism. Plant Omics..

[31] Zouari M (2019). Osmoprotectant-Mediated Abiotic Stress Tolerance in Plants 99–121.

[32] Silva FLB (2018). Proline accumulation induces the production of total phenolics in transgenic tobacco plants under water deficit without increasing the G6PDH activity. Theor. Exp. Plant Physiol..

[33] Zali AG, Ehsanzadeh P (2018). Exogenous proline improves osmoregulation, physiological functions, essential oil, and seed yield of fennel. Ind. Crops Prod..

[34] Aggarwal M (2011). Exogenous proline application reduces phytotoxic effects of selenium by minimising oxidative stress and improves growth in bean (*Phaseolus vulgaris* L.) seedlings. Biol. Trace Elem. Res..

[35] Chen Z (2021). Genome-wide analysis of terpene synthase gene family in Mentha longifolia and catalytic activity analysis of a single terpene synthase. Genes.

[36] Alonso-Ramírez A (2009). Evidence for a role of gibberellins in salicylic acid-modulated early plant responses to abiotic stress in *Arabidopsis* seeds. Plant Physiol..

[37] Emamverdian A, Ding Y, Mokhberdoran F (2020). The role of salicylic acid and gibberellin signaling in plant responses to abiotic stress with an emphasis on heavy metals. Plant Signal. Behav..

[38] Akula R, Ravishankar GA (2011). Influence of abiotic stress signals on secondary metabolites in plants. Plant Signal. Behav..

[39] Aftab T (2019). A review of medicinal and aromatic plants and their secondary metabolites status under abiotic stress. J. Med. Plant..

[40] Shaw JJ (2015). Identification of a fungal 1, 8-cineole synthase from Hypoxylon sp. with specificity determinants in common with the plant synthases. J. Biol. Chem..

[41] Yauk YK (2015). The O-methyltransferase gene MdoOMT 1 is required for biosynthesis of methylated phenylpropenes in ripe apple fruit. Plant J..

[42] Melo CR (2020). Synergistic effect of aromatic plant essential oils on the ant *Acromyrmex balzani* (Hymenoptera: Formicidae) and antifungal activity on its symbiotic fungus *Leucoagaricus gongylophorus* (Agaricales: Agaricaceae). Environ. Sci. Pollut. Res..

[43] Tarraf W (2015). Effects of mycorrhiza on growth and essential oil production in selected aromatic plants Ital. J. Agron..

[44] Linghu K-G (2019). 1, 8-Cineole ameliorates LPS-Induced vascular endothelium dysfunction in mice via PPAR-γ dependent regulation of NF-κB. Front. Pharmacol..

[45] Vosoughi N, Gomarian M, Ghasemi Pirbalouti A, Khaghani S, Malekpoor F (2020). The effects of water deficit stress and chitosan on cineole synthase gene expression and 1, 8-cineole content in sage (*Salvia officinalis*). Novin Genet. Sci..

[46] Bouwmeester HJ, Gershenzon J, Konings MCJM, Croteau R (1998). Biosynthesis of the monoterpenes limonene and carvone in the fruit of caraway: I: Demonstration of enzyme activities and their changes with development. Plant Physiol..

[47] Huchelmann A (2014). S-Carvone suppresses cellulase-induced capsidiol production in *Nicotiana tabacum* by interfering with protein isoprenylation. Plant Physiol..

[48] Ebadollahi A (2020). Estragole-rich essential oil of summer savory (*Satureja hortensis* L.) as an eco-friendly alternative to the synthetic insecticides in management of two stored-products insect pests. Acta Agric. Slov..

[49] Xie Y, Onik JC, Hu X, Duan Y, Lin Q (2018). Effects of (S)-carvone and gibberellin on sugar accumulation in potatoes during low temperature storage. Molecules.

[50] Chen F (2004). Characterization of a root-specific *Arabidopsis* terpene synthase responsible for the formation of the volatile monoterpene 1, 8-cineole. Plant Physiol..

[51] MetaCyc. Pathway: (4R)-carvone biosynthesis. https://biocyc.org/META/NEW-IMAGE?type=PATHWAY&object=PWY-5928 (2008).

[52] Wu X (2021). Biosynthesis of pinene in purple non-sulfur photosynthetic bacteria. Microb. Cell Factories..

[53] Majdi M, Malekzadeh-Mashhady A, Maroufi A, Crocoll C (2017). Tissue-specific gene-expression patterns of genes associated with thymol/carvacrol biosynthesis in thyme (*Thymus vulgaris* L.) and their differential changes upon treatment with abiotic elicitors. Plant Physiol. Biochem..

[54] Jordán MJ, Tandon K, Shaw PE, Goodner KL (2001). Aromatic profile of aqueous banana essence and banana fruit by gas chromatography−mass spectrometry (GC-MS) and gas chromatography−olfactometry (GC-O). J. Agric. Food Chem..

[55] Aubert C, Pitrat M (2006). Volatile compounds in the skin and pulp of Queen Anne's pocket melon. J. Agric. Food Chem..

[56] Ortiz-Serrano P, Gil JV (2010). Quantitative comparison of free and bound volatiles of two commercial tomato cultivars (*Solanum lycopersicum* L.) during ripening. J. Agric. Food Chem..

[57] Aragüez I (2013). Eugenol production in achenes and receptacles of strawberry fruits is catalyzed by synthases exhibiting distinct kinetics. Plant Physiol..

[58] Gross M (2002). Biosynthesis of estragole and t-anethole in bitter fennel (*Foeniculum vulgare* Mill. var. vulgare) chemotypes. Changes in SAM: Phenylpropene O-methyltransferase activities during development. Plant Sci..

[59] Dehsheikh AB (2020). Monoterpenes: Essential oil components with valuable features. Mini. Rev. Med. Chem..

