# Supplementary material for: Alternate gene expression profiling of monoterpenes in Hymenocrater longiflorus as a novel pharmaceutical plant under water deficit
Source: Sci Rep. 2022 Mar 8;12:4084. doi: 10.1038/s41598-022-08062-x (PMC8904481; doi:10.1038/s41598-022-08062-x)
Supplement: Supplementary file 4 — Supplementary Information 4. [file 41598_2022_8062_MOESM4_ESM.zip › RExpression/html/00Index.html]

R: Calculating the relative expression in RT-PCR

# Calculating the relative expression in RT-PCR

---

## Documentation for package ‘RExpression’ version 1.0.1

- DESCRIPTION file.

## Help Pages

|  |  |
| --- | --- |
| hello | Hello, World! |
