# Supplementary material for: Alternate gene expression profiling of monoterpenes in Hymenocrater longiflorus as a novel pharmaceutical plant under water deficit
Source: Sci Rep. 2022 Mar 8;12:4084. doi: 10.1038/s41598-022-08062-x (PMC8904481; doi:10.1038/s41598-022-08062-x)
Supplement: Supplementary file 4 — Supplementary Information 4. [file 41598_2022_8062_MOESM4_ESM.zip › RExpression/html/hello.html]

R: Hello, World!

|  |  |
| --- | --- |
| hello {RExpression} | R Documentation |

## Hello, World!

### Description

Prints 'Hello, world!'.

### Usage

```
hello()
```

### Examples

```
hello()
```

---

[Package *RExpression* version 1.0.1 Index]
