# Supplementary material for: Alternate gene expression profiling of monoterpenes in Hymenocrater longiflorus as a novel pharmaceutical plant under water deficit
Source: Sci Rep. 2022 Mar 8;12:4084. doi: 10.1038/s41598-022-08062-x (PMC8904481; doi:10.1038/s41598-022-08062-x)
Supplement: Supplementary file 5 — Supplementary Tables. [file 41598_2022_8062_MOESM5_ESM.docx]

| Supplementary Table S1. Primer sequences used for differential expression of the selected genes in surahalala | |
| --- | --- |
| Gene | Sequence |
| TPS2 | F 5՝- AGAAACTTTTAGAACTAGC -3՝ |
|  | R 5՝- CATCGACGTATTTGGACAGGAC -3՝ |
| TPS1 (thy) | F 5՝- ATGGCTACTTAGCCCATGCAAGTGTCC -3՝ |
|  | R 5՝- CACGGATATCTCGAAAATAAGGCCTCCC -3՝ |
| OMT (estragole) | F 5՝- GTGGAGTTGAGTGTTGAGGT -3՝ |
|  | R 5՝- CAGTTGTTGTGAGGGAATGC -3՝ |
| GDH1 (Citronellol) | F 5՝- ATCATCCGCTGGCCTGAAAA -3՝ |
|  | R 5՝- GGGAAGGCCAACCACGATAA -3՝ |
| L3H (carv) | F 5՝- GCTGACTTGTTCCCCTCCTC -3՝ |
|  | R 5՝- CATCCACACTCGTCTTCCCC -3՝ |
| TPS27 (Cin) | F 5՝- ACAGCTACCAAGTTCACGGG -3՝ |
|  | R 5՝- CGAGTGCTGTTTCCGGTAGT -3՝ |
| Elongation factor 1 | F 5՝-CCAAGATTGACAGGAGGTCTGGAAA-3՝ |
|  | R 5՝-ATGCAGGTATGGTGAAGATGCTTCC-3՝ |
| Actin | F 5՝- TGACCGAATGAGCAAGGAAATTACT -3՝ |
|  | R 5՝- TACTCAGCTTTGGCAATCCACATC -3՝ |

| Supplementary Table S2. Mean comparison for expression rate of the genes in surahalala proportional to the EF1 as internal control | | | | | | | | |
| --- | --- | --- | --- | --- | --- | --- | --- | --- |
| PEG | Proline | TPS27 | L3H | TPS2 | TPS1 | OMT | GDH1 |  |
| 0 | 0 | 1±0e | 1±0gh | 1±0c | 1±0i | 1±0hi | 1±0h |  |
| 0 | 5 | 1.57±0.025e | 1.44±0.022g | 0.5±0.011e | 1.88±0.029h | 1.5±0.025ghi | 3.95±0.1fg | |
| 0 | 10 | 0.6±0.006e | 1.2±0.013g | 0.13±0.002hi | 2.43±0.022gh | 0.16±0.002i | 10.26±0.143e | |
| 0 | 15 | 4.45±0.043d | 3.94±0.047e | 0.33±0.006f | 4.69±0.068f | 0.56±0.006i | 10.95±0.264e | |
| 0 | 20 | 0.31±0.004e | 0.28±0.004hi | 0.05±0.001i | 0.19±0.002j | 0.5±0.007i | 0.32±0.004h | |
| 10 | 0 | 7.57±0.109c | 6.03±0.091d | 2.67±0.06a | 4.85±0.078f | 7.57±0.109e | 4.75±0.127fg | |
| 10 | 5 | 6.09±0.117cd | 2.95±0.066f | 0.39±0.007f | 2.16±0.028gh | 4.31±0.083f | 4.59±0.073fg | |
| 10 | 10 | 38.48±0.749b | 22.98±0.442a | 0.67±0.018d | 13.77±0.262b | 10.31±0.201d | 57.85±1.801a | |
| 10 | 15 | 54.77±0.835a | 9.65±0.106c | 0.3±0.004fg | 12.05±0.117c | 3.19±0.049fg | 28.35±0.353b | |
| 10 | 20 | 38.8±0.771b | 19.21±0.378b | 1.23±0.034b | 6.4±0.122e | 63.03±1.252a | 10.92±0.341e | |
| 20 | 0 | 0.05±0e | 0.04±0i | 0.07±0.001i | 0.27±0.002ij | 1.25±0.016hi | 0.1±0.001h | |
| 20 | 5 | 0.02±0e | 0.02±0i | 0.13±0.002hi | 2.7±0.03g | 2.76±0.039fgh | 2.16±0.04gh | |
| 20 | 10 | 0.29±0.003e | 0.19±0.002i | 0.2±0.002h | 9.03±0.082d | 20.07±0.216c | 14.5±0.177d | |
| 20 | 15 | 0.21±0.003e | 0.13±0.002i | 0.21±0.005gh | 26.11±0.479a | 3.11±0.04fg | 23.07±0.694c | |
| 20 | 20 | 0.12±0.001e | 0.15±0.002i | 0.5±0.009e | 8.61±0.116d | 48.37±0.482b | 5.58±0.125f | |
| Mean ± Standard error of mean | | | |  |  |  |  |  |
| Means with the letter(s) are significantly different (LSD 5%) | | | | | | |  |  |

| Supplementary Table S3. Mean comparison for expression rate of the genes in surahalala proportional to actin as internal control | | | | | | | |
| --- | --- | --- | --- | --- | --- | --- | --- |
| PEG | Prolinee | TPS27 | L3H | TPS2 | TPS1 | OMT | GDH1 |
| 0 | 0 | 1±0de | 1±0e | 1±0b | 1±0fg | 1±0f | 1±0ij |
| 0 | 5 | 0.12±0.002e | 0.12±0.001g | 0.04±0.001h | 0.16±0.002i | 0.09±0.001f | 0.34±0.004j |
| 0 | 10 | 0.18±0.003e | 0.34±0.005fg | 0.04±0.001h | 0.65±0.009gh | 0.05±0.001f | 2.8±0.041ef |
| 0 | 15 | 2.54±0.077cde | 0.82±0.014ef | 0.06±0.001h | 0.65±0.01gh | 0.15±0.003f | 1.55±0.025ghi |
| 0 | 20 | 4.24±0.134cd | 3.47±0.065c | 0.57±0.011c | 1.86±0.033e | 7.02±0.135cd | 3.24±0.058e |
| 10 | 0 | 5.23±0.163c | 3.59±0.067c | 1.47±0.027a | 2.42±0.045d | 5.33±0.101d | 2.42±0.045efg |
| 10 | 5 | 2.22±0.061cde | 1.26±0.016e | 0.18±0.002f | 1.16±0.012f | 1.6±0.026ef | 2.49±0.025efg |
| 10 | 10 | 22.3±0.416b | 13.47±0.247b | 0.4±0.007d | 8.19±0.145c | 5.94±0.136cd | 35.12±0.621a |
| 10 | 15 | 134.02±2.634a | 17.12±0.328a | 0.4±0.007d | 11.21±0.197a | 7.76±0.185c | 26.67±0.469b |
| 10 | 20 | 4.17±0.083cd | 2.08±0.041d | 0.14±0.003g | 0.71±0.013gh | 6.73±0.161cd | 1.23±0.022hij |
| 20 | 0 | 0.01±0e | 0.01±0g | 0.16±0.002fg | 0.58±0.008h | 3.23±0.053e | 0.22±0.003j |
| 20 | 5 | 0.01±0e | 0.01±0g | 0.05±0.001h | 0.83±0.015fgh | 1.78±0.035ef | 0.67±0.012ij |
| 20 | 10 | 0.55±0.01e | 0.3±0.005fg | 0.28±0.005e | 10.7±0.173b | 37.67±0.844b | 17.39±0.282c |
| 20 | 15 | 0.05±0.001e | 0.02±0g | 0.02±0h | 2.31±0.023d | 0.73±0.011f | 2.08±0.021fgh |
| 20 | 20 | 0.18±0.005e | 0.19±0.003g | 0.56±0.008c | 8.39±0.105c | 76.99±1.262a | 5.53±0.069d |
| Mean ± Standard error of mean | | | |  |  |  |  |
| Means with the letter(s) are significantly different (LSD 5%) | | | | | | |  |

| Supplementary Table S4. Canonical correlation extracted indices and standard scores of gene expression versus measured features in surahalala | | | | | | |
| --- | --- | --- | --- | --- | --- | --- |
| Index N. | CC1 | CC2 | CC3 | CC4 | CC5 | CC6 |
| Value | 0.998 | 0.998 | 0.997 | 0.933 | 0.838 | 0.414 |
|  |  |  |  |  |  |  |
| Gene | CC1 | CC2 | CC3 | CC4 | CC5 | CC6 |
| TPS27 | 0.016 | 0.325 | -0.883 | -0.152 | -0.224 | -0.041 |
| L3H | 0.294 | 0.518 | -0.541 | 0.278 | -0.394 | -0.081 |
| TPS2 | -0.378 | -0.065 | 0.101 | 0.225 | -0.346 | -0.324 |
| TPS1 | -0.294 | 0.793 | -0.363 | 0.152 | 0.293 | -0.008 |
| OMT | 0.295 | 0.447 | 0.194 | -0.286 | 0.372 | -0.256 |
| GDH1 | 0.164 | 0.577 | -0.587 | 0.503 | 0.028 | 0.005 |
|  |  |  |  |  |  |  |
| Feature | CC1 | CC2 | CC3 | CC4 | CC5 | CC6 |
| Cinle | -0.241 | 0.105 | -0.904 | 0.052 | -0.074 | -0.09 |
| Carv | 0.214 | -0.174 | -0.355 | 0.527 | -0.104 | 0.043 |
| Alph_P | -0.111 | 0.081 | 0.114 | -0.348 | 0.533 | -0.175 |
| Thy | 0.183 | 0.456 | -0.618 | -0.223 | -0.381 | -0.108 |
| Est | 0.062 | -0.33 | -0.001 | 0.027 | 0.2 | -0.034 |
| Bet_C | -0.013 | 0.636 | -0.694 | -0.012 | -0.193 | -0.15 |
| SOD | 0.204 | 0.039 | 0.214 | -0.361 | 0.494 | -0.141 |
| PRO | 0.036 | 0.708 | -0.245 | 0.115 | 0.067 | -0.117 |
| H2O2 | -0.034 | 0.429 | 0.345 | -0.3 | 0.27 | -0.139 |
| SFW | 0.178 | -0.424 | -0.099 | 0.025 | -0.315 | 0.554 |
| RFW | -0.299 | -0.346 | -0.543 | 0.348 | -0.397 | -0.252 |
